# Factors Contributing to Decline in Foodborne Disease Outbreak Reports, United States

**DOI:** 10.3201/eid2009.140044

**Published:** 2014-09

**Authors:** Maho Imanishi, Karunya Manikonda, Bhavini P. Murthy, L. Hannah Gould

**Affiliations:** Centers for Disease Control and Prevention, Atlanta, Georgia, USA

**Keywords:** foodborne diseases, outbreaks, surveillance, United States, enteric infections

## Abstract

The number of foodborne disease outbreaks reported in the United States declined substantially in 2009, when the surveillance system transitioned from reporting only foodborne disease outbreaks to reporting all enteric disease outbreaks. A 2013 survey found that some outbreaks that would have been previously reported as foodborne are now reported as having other transmission modes.

Since 1973, the Centers for Disease Control and Prevention (CDC) has collected data on foodborne disease outbreaks submitted by all states, the District of Columbia, and US territories through the Foodborne Disease Outbreak Surveillance System. In 2009, existing foodborne and waterborne disease outbreak surveillance systems were transitioned to an enhanced reporting platform, the National Outbreak Reporting System (NORS), which also collects reports of enteric disease outbreaks transmitted through person-to-person contact, contact with animals, environmental contamination, and indeterminate means (*1*). A new electronic reporting form and data entry interface were also introduced. In 2009, the number of reported foodborne disease outbreaks declined 32% compared with the mean of the preceding 5 years (*2*); the number also remained below the pre-2009 average during 2010–2012 (*2*,*3*) (Figure). The decline was largely observed among outbreaks attributed to norovirus (Figure), which can be transmitted through many routes: in comparison, the number of outbreaks attributed to *Salmonella* spp., which is usually transmitted through food, remained relatively constant (*1*,*2*).

**Figure:**
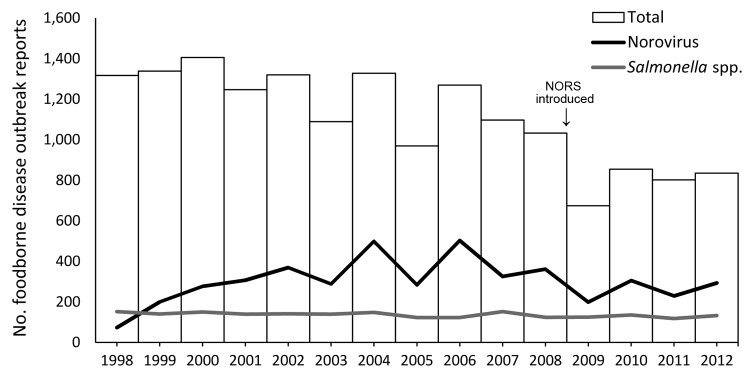
Total number of foodborne disease outbreaks and number caused by norovirus and *Salmonella* spp. as reported to the Foodborne Disease Outbreak Surveillance System, United States, 1998–2012. Data current as of April 22, 2014.

We considered 3 possible reasons for the decline in the number of reported foodborne disease outbreaks: 1) classification of some outbreaks that previously would have been reported as foodborne as caused by another modeof transmission; 2) technical issues associated with the introduction of the new system; and 3) staffing and resource limitations related to the influenza A(H1N1)pdm09 virus pandemic. Clarification of how these factors might have affected reporting would provide accurate conclusions about trends in foodborne disease outbreaks. In 2013, we conducted a survey to identify possible reasons for the decline in the number of foodborne disease outbreak reports that started in 2009.

## The Study

In January 2013, we conducted a voluntary, anonymous, internet-based survey of public health officials who are responsible for entering foodborne disease outbreak data into NORS at US state and territory health departments. The survey contained 33 questions in multiple choice, rating scale, or text formats. The questions asked about reporting procedures that might influence data quality and completeness, challenges and practices when determining the mode of transmission for each outbreak, the usability of the online reporting interface, and resource limitations.

Of the 133 public health officials in 56 jurisdictions who received the link to the survey, 50 (38%) from 39 (70%) jurisdictions completed the survey in whole or in part. The denominator varied for different questions because of nonresponse and exclusion of responses when “don’t know” was selected. Also, some respondents had not used the previous reporting system. Most respondents (36/43, 84%) assigned a high priority to entering foodborne disease outbreak data, rating outbreak reporting as 4 or 5 on a scale of 1 to 5, where 1 indicated low priority and 5 high priority. Similarly, 38/47 (81%) of respondents reported that 90%–100% of foodborne disease outbreaks investigated in their health departments were entered into NORS; 5/47 (11%) reported entering <50% of outbreaks.

The survey included 1 question to determine whether respondents had experienced difficulties identifying the primary mode of transmission for some outbreaks and 1 question to understand which modes of transmission they found difficult to distinguish from foodborne transmission. Many respondents (35/47, 74%) reported sometimes having difficulties in determining an outbreak’s primary mode of transmission. More than half (26/47, 55%) of respondents reported that, since 2009, they had used the newly established category of indeterminate/other/unknown to report an outbreak for which the mode of transmission was not clear. Twenty (80%) of 25 respondents reported that they had experienced difficulty distinguishing between foodborne and person-to-person transmission (Table). In comparison, determining whether an outbreak was caused by transmission of the infectious agent through food or by animal contact was a problem for only 6 of 21 (29%) respondents. Respondents who reported difficulty distinguishing between foodborne and another mode of transmission were asked if they would have reported the outbreak as foodborne to the previous reporting system (before 2009). Most respondents indicated that an outbreak was very likely or likely to have been reported to the previous system as a foodborne disease outbreak if there was a problem determining whether an outbreak was caused by foodborne or person-to-person transmission (15/20 respondents, 75%); by foodborne or environmental contamination (8/11 respondents, 73%); or if a specific mode of transmission could not be determined with confidence (13/19 respondents, 68%).

**Table:** Number of survey respondents reporting difficulty in distinguishing between foodborne disease outbreaks and outbreaks caused by other modes of transmission in the National Outbreak Reporting System, United States, 2013

Mode of transmission	No. (%) respondents	
	Experienced difficulty in distinguishing outbreak type	If experienced difficulty, likely reported as foodborne to previous system
Foodborne vs. person-to-person, n = 25	20 (80)	15 (75)
Foodborne vs. indeterminate/other/unknown, n = 25	19 (76)	13 (68)
Foodborne vs. environmental contamination, n = 22	11 (50)	8 (73)
Foodborne vs. water, n = 23	8 (35)	2 (25)
Foodborne vs. animal contact, n = 21	6 (29)	1 (17)

Regarding usability of the NORS reporting interface, most respondents (36/37, 97%) reported that usability of the new interface was the same as or better than that of the previous system. Most respondents (26/31, 84%), reported that technical issues with the NORS system did not prevent them from entering outbreak reports; only 2 respondents reported that >10% of outbreaks were not entered because of technical issues. When asked if their health departments experienced decreased resources available to work on foodborne disease outbreaks in 2009 while dealing with influenza A(H1N1)pdm09 virus, 19 (57%) of 35 respondents reported decreased resources for foodborne disease outbreak investigations; 14 (44%) of 32 respondents reported decreased resources for outbreak detection (e.g., laboratory capacity); and 14 (38%) of 37 respondents reported decreased resources for outbreak data entry and reporting.

## Conclusions

Clarifying the factors that affect foodborne disease outbreak surveillance enables accurate interpretion of observed changes over time. The findings of this survey suggest that the large decline in the number of foodborne disease outbreaks reported in 2009 was likely a combined result of the following: 1) a surveillance artifact, in that some outbreaks previously reported as foodborne are now attributed to other modes of transmission; and 2) limited availability of resources to detect, investigate, and report foodborne disease outbreaks during the influenza A(H1N1)pdm09 pandemic. The total number of outbreaks reported increased after 2009 but remained below pre-2009 numbers, which suggests that the effect of the surveillance artifact is persistent and that outbreaks are now being more accurately categorized by mode of transmission. Although we hypothesized that technical issues with the new reporting interface might have affected reporting, this explanation appears less likely.

Limitations of the survey included the length of time between the transition to NORS and the survey, which meant that some survey participants who are current NORS users had not used the previous reporting system or worked on foodborne disease outbreaks in 2009. This limitation explains the low number of responses to survey questions that required knowledge of practices before 2009. Also, the overall response rate for the survey was low. Possible explanations include staffing and resource limitations, but the survey was voluntary and anonymous, and no follow-up efforts were made to determine reasons for the low response rate. In addition, because some jurisdictions have >1 reporting administrator, personnel in some jurisdictions may have compiled a single response. On the other hand, the experiences of some health departments that did not compile responses might have been overrepresented. Further, the survey was not designed to measure the proportion of reported outbreaks affected by the introduction of NORS. Other potential reasons for the decline in the number of reported foodborne disease outbreaks, such as resource limitations and loss of public health positions resulting from budget cuts during the recession (*4*), were not explored.

In summary, the results of this survey provide unique insights into the decline in the number of foodborne outbreak reports submitted in 2009 and thereafter. NORS provides more comprehensive surveillance of outbreaks and a better understanding of the epidemiology of pathogens with multiple transmission pathways (*1*). These findings may be useful to improve guidance and training for outbreak reporting, particularly in reporting of the mode of transmission when multiple pathways exist for a pathogen. 
